# Correction to ‘Predictive value of biomarker signatures for suicide risk in hospitalised patients with major depressive disorders: A multicentre study in Shanghai’

**DOI:** 10.1002/gps3.70068

**Published:** 2026-10-08

Zhu E, Wang J, Cai Z, Zhou G, Li C, Chen F, et al. Predictive value of biomarker signatures for suicide risk in hospitalised patients with major depressive disorders: a multicentre study in Shanghai. *Gen Psychiatr*. 2025;38:e101957. https://doi.org/10.1136/gpsych‐2024‐101957.

1. In the originally published HTML version of this article, ‘Deceased Dr Zisheng Ai’ was incorrect.

‘Deceased Dr Zisheng Ai’ should be deleted.

We apologise for this error.

